# Uncontrolled Eating Through the Lens of Mentalization and Emotional Eating: The Moderating Role of Food Addiction

**DOI:** 10.3390/nu17203233

**Published:** 2025-10-15

**Authors:** Alessandro Alberto Rossi, Andrea Tagliagambe, Anna Scuderi, Laura Dalla Ragione, Stefania Mannarini

**Affiliations:** 1 Department of Philosophy, Sociology, Education, and Applied Psychology, Section of Applied Psychology, University of Padua, 35131 Padua, Italy; 2 Center for Intervention and Research on Family Studies—CIRF—Department of Philosophy, Sociology, Education, and Applied Psychology, Section of Applied Psychology, University of Padua, 35131 Padua, Italy; 3 Residence Cabrini DCA, 54027 Pontremoli, Italy; 4 Food Science and Human Nutrition Unit, University Campus Biomedico of Rome, 00128 Rome, Italy

**Keywords:** food addiction, reflective functioning, mentalization, emotional eating, uncontrolled eating, binge eating, eating addiction

## Abstract

**Background**. The literature suggests that deficits in mentalization, operationalized as reflective functioning, are associated with emotional and behavioral dysregulation, including emotional eating and uncontrolled eating. These eating behaviors may be intensified by food addiction, yet its moderating role within this framework has not been thoroughly investigated. This study examined whether the relationship between deficits in reflective functioning and uncontrolled eating is mediated by emotional eating, and whether food addiction diagnosis moderates this pathway. **Methods**. A cross-sectional survey was administered to 559 adults from the general population. Participants completed self-report measures assessing reflective functioning (RFQ-8), emotional and uncontrolled eating (TFEQ-R-18), and food addiction (YFAS 2.0). A moderated mediation model was tested using conditional process analysis with 10,000 bootstrap resamples. **Results**. Deficits in reflective functioning were positively associated with emotional eating (β = 0.155, *p* < 0.001), which in turn were associated with uncontrolled eating (β = 1.314, *p* < 0.001). Food addiction diagnosis significantly moderated the relationship between emotional eating and uncontrolled eating (β = 0.744, *p* < 0.001). Specifically, individuals with food addiction exhibited a stronger association between emotional eating and uncontrolled eating compared to those without food addiction. The indirect effect from reflective functioning to uncontrolled eating via emotional eating was significantly stronger among individuals with food addiction than those without. The overall model explained 57.3% of the variance in uncontrolled eating. **Conclusions**. Food addiction diagnosis amplifies the pathway from emotional eating to uncontrolled eating, originating from deficits in reflective functioning. These findings highlight the clinical importance of targeting mentalization processes and emotional eating in interventions for disordered eating behaviors, particularly among individuals with food addiction.

## 1. Introduction

Disordered eating behaviors involving excessive food intake encompass a spectrum of atypical eating patterns characterized by dysfunctional responses to food-related cues, which can lead to harmful consequences, such as overweight, obesity and cardiovascular issues [1,2]. Often, these maladaptive eating patterns appear to be linked by certain recurring transdiagnostic factors (e.g., emotion dysregulation, impulsivity, childhood trauma) [3,4], as well as by behaviors, signs, and symptoms associated with a possible addiction to food and eating [5]. Research on eating behaviors has identified various forms of excessive consumption, ranging from persistent, repetitive, and compulsive grazing to episodes of overeating and binge eating [5,6,7,8].

Scientific studies highlighted that the development and maintenance of disordered eating behaviors are multifaceted, resulting from a complex interplay of genetic, behavioral, social, and psychological factors [9,10,11,12]: the literature has identified numerous risk factors and triggering cues [13,14], although the nature of their interrelationships and their specific contributions remain not fully understood.

According to infancy research and the attachment theory, previous studies have identified several risk factors for disordered eating behaviors, particularly early traumatic experiences such as emotional maltreatment [15,16,17,18,19] and insecure attachment to primary caregivers [15,20,21]. These early traumatic adversities are consistently linked to a heightened vulnerability to disordered eating patterns [15,17]. Individuals exposed to such experiences often develop attachment insecurities, which are associated with both eating-related difficulties [20,22] and broader psychopathology [23,24]. The literature indicates that childhood maltreatment may disrupt the attachment system [25,26], leading to impaired self-regulation and a fragmented sense of self due to emotionally mis-attuned caregiver relationships (i.e., insecure attachment) [27,28,29,30,31]—that would normally allow the child to use the parent as a secure base. Furthermore, these relational disruptions can hinder the development of core interpersonal capacities, including mentalization [25,32,33].

The construct of mentalization, often referred to as reflective functioning, is closely tied to early child–caregiver relationships [34,35]. Reflective functioning refers to the ability to interpret and make sense of one’s own behavior and that of others by attributing it to underlying mental states, including emotions, thoughts, intentions, and desires [36]. More specifically, reflective functioning involves understanding human actions by attributing them to underlying mental states such as thoughts, beliefs, feelings, and emotions [37,38]. This capacity enables individuals to ascribe meaning to others’ behaviors and experiences without relying entirely on external validation or cues [36,37].

However, when reflective functioning is underdeveloped or impaired, this foundational interpersonal capacity remains compromised [37]. Reflective functioning, when dysfunctional, can be seen as an automatic and unconscious process that negatively affects a person’s ability to interpret and regulate behavior [39], undermining the individual’s capacity to inhibit impulsive or dysregulated responses, particularly in emotionally charged situations [40]. Uncertain or impaired mentalization is often associated with poor emotion regulation difficulties with self-monitoring, impulse control, and a disrupted sense of self-agency [37,41,42,43]. A compromised reflective function often results in a rigid, distorted understanding of mental states leading individuals experiencing reality in a different, prementalized manner [15,25,44]. In such states, people experience a breakdown between internal feelings and external reality [25,44], especially during times of distress, making it difficult to distinguish between emotional experiences and actual events [28,40]. In these cases, internal states are perceived as disorganizing and overwhelming [28,37].

Consequently, individuals with impaired reflective functioning may seek to defend themselves from these distressing or threatening emotions [39,45,46] by relying on maladaptive coping strategies [41,42,47] that provide an immediate relief [32,48]—such as emotionally driven, compulsive, disordered eating behaviors [7,49,50].

Previous research has identified failures in reflective functioning as a key feature in individuals with disordered eating behaviors [15,17,51,52], including emotional eating, overeating, and loss of control over eating. So, in response to emotionally distressing or psychologically dysregulating experiences, these individuals may turn toward more tangible or bodily-based coping strategies *(*e.g., focusing on their physical body) [15] or external regulators such as food [51,53], in a compensatory effort to manage psychological and emotional discomfort and restore a sense of balance.

Thus, when faced with internal states perceived as aversive—such as anxiety, anger, intrusive thoughts, or feelings of uncontrollability—individuals with low reflective functioning may resort to impulsive and emotionally-driven eating behaviors.

Indeed, as levels of psychological stress or arousal increase, these individuals tend to rely on more primitive, unconscious, and automatic regulation strategies that promise immediate relief [32,48]—such as substance use or compulsive, emotionally driven eating behaviors. In these situations, the urge to escape intolerable emotional states can culminate in emotional eating [54,55,56] that consists in a powerful, overwhelming compulsion to eat (see e.g., [57,58]). Consequently, the more this emotion regulation strategy is effective in proving relief to discomfort, the more the individual will rely on it, and the more this process will tend to be reinforced and maintained [17]. This, in turn, may potentially lead the individual to develop a dependence/addiction on this external regulator (in this case, food) for emotion regulation, reinforcing dysfunctional emotional eating behaviors.

However, when highly stressful or traumatic events occur, individuals may feel engulfed by unbearable affective states and milder emotional regulation strategies—like emotional eating—prove to be insufficient [25,46]. As a result, they may turn to more intense and maladaptive responses—such as binge eating or compulsive, uncontrolled eating—in an attempt to fully suppress or numb the intense negative emotional experience [49,59,60,61]. These uncontrolled eating behaviors differ from emotional eating in their severity because they imply the loss of control over eating.

However, the relationship from emotional eating to uncontrolled eating may be influenced by another key construct, food addiction (FA), which could foster engagement in dysregulated eating behaviors [49] and which in recent years has gained increasing attention in both clinical practice and research related to eating disorders and dysfunctional eating behaviors [62,63,64,65]. Recent research has highlighted that certain individuals may develop signs of addiction to highly palatable and processed foods (HPFs) high in sugar, fat, and salt [66,67,68], which are particularly activating for brain circuits and neurotransmitters involved in reward and pleasure, similar to brain circuitry implicated in substance addiction [69,70,71,72].

Individuals diagnosed with FA may exhibit the core behavioral patterns typically associated with addiction—such as tolerance, withdrawal, and craving—which in turn may foster, reinforce, and maintain overeating and uncontrolled food intake behaviors [17,49]. FA appears to be a highly complex and multifaceted construct, it encompasses several facets, including impulsivity, emotional dysregulation, and poor dietary control [60,73,74,75]. FA also appears to have a transdiagnostic nature; it is observed in individuals with anorexia nervosa, bulimia nervosa, and binge eating disorder, as well as in clinical populations and in the general population [76]. It should also be acknowledged that food addiction is not confined to evident episodes of binge eating or overeating; it may also appear in more subtle and ongoing behaviors, such as grazing [6,7].

However, FA is still relatively poorly understood, as its etiology remains largely unclear [5]. Some recent studies have linked FA to experiences of physical, sexual, and emotional trauma during childhood [17,77,78,79]. Furthermore, a recent study by Rossi et al. (2025) has also associated FA with deficits in individuals’ reflective functioning [17].

Studies show that individuals diagnosed with FA—both from clinical samples and the general population—tend to exhibit a greater number and intensity of dysregulated eating behaviors compared to those without a diagnosis [80].

Relying on this evidence, it is therefore plausible to hypothesize that the strength of the association between emotional eating and uncontrolled eating may be moderated by the presence of an FA diagnosis. In other words, the strength of the relationship between emotional eating and uncontrolled eating may be moderated by whether or not the individual is diagnosed with FA.

However, this hypothesis still lacks robust empirical validation. Indeed, despite the extensive literature, the relationship between maladaptive eating behaviors and FA remains not fully understood—especially when considering early experiences with caregivers during childhood. Moreover, previous studies have often overlooked the underlying mechanisms that might link psychological factors rooted in early development (such as mentalization/reflective functioning), uncontrolled eating behaviors, and FA.

Consequently, based on these theoretical premises, the present research aims to examine a moderated mediation model in which deficits in reflective functioning (X) predict uncontrolled eating behaviors (Y) through emotional eating (M), with the presence of a FA diagnosis (W) moderating the whole model—this being the primary hypothesis. This model seeks to clarify how impaired mentalization may lead to maladaptive eating patterns and explore the amplifying effect of FA on these relationship.

**H1.**  *Deficits in reflective functioning, dysfunctional eating behaviors (emotional eating and uncontrolled eating), and FA diagnosis are positively correlated;*

**H2.**  *Individuals diagnosed with FA will show higher levels of reflective functioning deficits, emotional eating and uncontrolled eating;*

**H3.**  *FA diagnosis will moderate the mediation model.*

In other words, this study aimed to examine whether the relationship from (deficits in) reflective functioning to uncontrolled eating is mediated by emotional eating and whether this mediation pathway is moderated by the presence of a FA diagnosis. Specifically, it was hypothesized that individuals with greater deficits in reflective functioning would show increased emotional eating behaviors, which would in turn predict higher levels of uncontrolled eating. Furthermore, it was expected that the presence of a FA diagnosis would strengthen the association between emotional eating and uncontrolled eating, thereby amplifying the indirect pathway from (deficits in) reflective functioning to uncontrolled eating behaviors. With exploratory aims, the moderating effect of FA was also tested from (deficits in) reflective functioning to emotional eating, and from (deficits in) reflective functioning to uncontrolled eating.

## 2. Methods and Materials

### 2.1. Procedure

Participants were recruited from the general population using a snowball sampling approach [81], primarily through social media platforms, in line with previous studies [50,73,82]. To be eligible, individuals had to meet the following criteria: (A) be 18 years of age or older, (B) have Italian as their native language, (C) provide fully completed responses, and (D) signed and submit an online informed consent form. Ethical approval for the study was granted by the Ethics Committee of the University of Padua (protocol number 3558).

### 2.2. Sample Size Determination

The required sample size was determined a priori using a Monte Carlo simulation conducted in R (v 4.3.2), following general recommendations for power analysis in path analysis according to Muthén and Muthén (2002) [83]. The model specified a moderated mediation structure, including conditional indirect effect depending on the level of a binary moderator (0 = no FA, 1 = presence of FA). In line with previous studies (e.g., [15,17,49]) and the guidelines of Cohen (1988), path coefficients were set to small-to-moderate standardized values [84]. A Type I error rate (α) of 0.05 was adopted, and the desired statistical power (1 − β) was set to 0.80 [84]. Datasets were generated and analyzed using the lavaan package in R. Results indicated that a minimum sample size of 350 participants would yield an estimated power of 0.82 to detect the moderated indirect effect.

### 2.3. Measures

A demographic questionnaire was administered to gather information on participants’ age, biological sex, educational attainment, marital status, and employment status. Participants were also asked to report whether they had received a diagnosis of an eating disorder (ED) and to provide their weight and height, which were used to compute their body mass index (BMI).

#### 2.3.1. Reflective Functioning Questionnaire 8 (RFQ-8)

The RFQ-8 [85] was utilized to measure participants’ mentalization operationalized as reflective functioning—i.e., their ability to understand their own and others’ mental states. This instrument includes 8 items rated on a 7-point Likert scale, from “completely disagree” (=1) to “completely agree” (=7), asking respondents to indicate their agreement with statements about mentalizing abilities. The RFQ-8 assesses two contrasting aspects: certainty and uncertainty about mental states. In this study, the uncertainty scale (RFQ8u) was used to capture deficits in mentalization/reflective functioning, with higher scores indicating greater impairment. The Italian version of the RFQ-8 [86] was employed, demonstrating good internal consistency: RFQ8u, McDonald’s omega = 0.791.

#### 2.3.2. The Three-Factor Eating Questionnaire Revised 18 (TFEQ-R-18)

The TFEQ-R-18 [87] was use to assess key cognitive and behavioral dimensions associated with eating behaviors: cognitive restraint (CR), uncontrolled eating (UE), and emotional eating (EE). It consists of 18 items, each rated on a 4-point Likert scale, where higher scores reflect greater tendencies in the respective domains. The CR subscale captures individuals’ efforts and preoccupations with regulating their food intake to control body weight and shape—consequently, since its content was not relevant to the aims of the present study, this subscale was not used for the statistical analyses. The EE subscale measures the tendency to eat in response to emotional states, whether positive or negative, such as distress or other negative emotions. The UE subscale, on the other hand, assesses the difficulty in maintaining control over eating behaviors, often leading to overeating episodes. For this research, the Italian adaptation of the TFEQ-R-18 was employed [88]. The internal consistency, evaluated through McDonald’s omega, was 0.870 for the EE subscale and 0.822 for the UE subscale.

#### 2.3.3. The Yale Food Addiction Scale 2.0 (YFAS 2.0)

The YFAS 2.0 [80,89] is a self-report instrument developed to assess the occurrence of FA symptoms over the previous 12 months. It includes 35 items rated on an 8-point Likert scale and is based on the DSM-5 criteria for substance use disorders (SUD) [62]. In particular, diagnostic criteria are: (A) consumed larger amount and for a longer period than intended; (B) much time/activity to obtain, use, recover; (C) social activities given up or reduced; (D) withdrawal symptoms; (E) Failure to fulfil major role obligation; (F) use despite knowledge of adverse consequences; (G) tolerance; (H) craving; (I) persistent desire or unsuccessful attempts to quit; (J) use in physically hazardous situations; (K) Social or interpersonal problems. FA can be evaluated through two scoring methods: (I) the symptom count, which tallies the number of criteria met, and (II) the diagnostic score, which classifies severity into No FA, mild, moderate, or severe FA depending on the level of impairment or distress. According to previous research [80], in this study, the aggregated diagnostic score was used. Thus, participants were classified as (A) non-food addicted (i.e., No FA = 0) or (B) food addicted (mild, moderate and severe FA = 1). The Italian version of the YFAS 2.0 [80,90] was used, showing good internal consistency: McDonald’s omega for categorical data = 0.875.

### 2.4. Statistical Analysis

Statistical analyses were performed with the R software (v. 4.3.2). The following packages were used: interactions, lavaan, lme4, process for R, psych, and tidyverse.

First (H1), the Pearson correlation coefficient (*r*) was computed to evaluate the strength of the relationships between variables [91,92] and interpreted using Cohen’s benchmarks [84]: *r* < 0.10, trivial; *r* from 0.10 to 0.30, small; *r* from 0.30 to 0.50, moderate; *r* > 0.50, large.

Second (H2), a Multivariate Analysis of Variance (MANOVA) was conducted to explore potential differences across FA diagnosis groups (No diagnosis of FA vs. FA Diagnosis—independent variable) on the three scales of the mediation model (i.e., reflective functioning, emotional eating, and uncontrolled eating—dependent variables) considered simultaneously. Wilks’ Lambda (Λ) was used to evaluate the overall multivariate effect [91,93] and interpreted using partial eta squared (η^2^_p_), classified as small (0.011–0.059), moderate (0.060–0.139), or large (>0.140). Effect sizes for bivariate comparisons were interpreted using Hedge’s g (*g*), classified as small (≈0.2), moderate (≈0.50), or large (≈0.80) [84].

**Figure 1:**
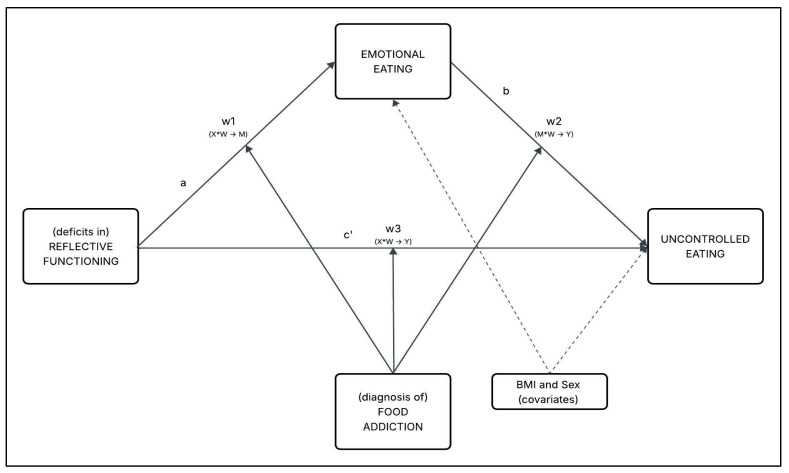
Path model–conceptual representation.

Third (H3), in line with previous studies, before testing the hypothesized conditional process model (moderated mediation), preliminary analyses were performed. Then, a conditional process analysis (moderated mediation; 10,000 bootstrap resamples) with observed variables was performed [92,94,95,96,97,98,99]. The maximum likelihood (ML) estimator was used to conduct the statistical analyses. Following previous studies, two analytical steps were carried out. *First*, a simple mediation model was specified: ‘(deficits in) reflective functioning’ (X) was hypothesized to influence ‘uncontrolled eating’ (Y) through ‘emotional eating’ (M). *Second*, a conditional process model was tested: while maintaining the same mediation structure, the path from ‘emotional eating’ (M) to ‘uncontrolled eating’ (Y) was moderated by ‘(diagnosis of) FA’ (W), a binary variable coded as absence (=0) versus presence (=1) of FA diagnosis [92,100,101,102] (see Figure 1).

The moderating effect of ‘(diagnosis of) FA’ (W) was examined by estimating the conditional effects at each level of the moderator (W = 0 and W = 1) within a single conditional process model, using 95% confidence intervals (95%CI) [92,95]. Given the categorical nature of the moderator, the analyses focused on comparing the magnitude and significance of the direct and indirect effects across the two groups. The interaction term between ‘emotional eating’ (M) and ‘(diagnosis of) food addiction’ (W) was specifically tested to assess whether the effects on ‘uncontrolled eating’ (Y) differed depending on the presence (W = 1) or absence (W = 0) of the FA diagnosis. Lastly, the index of moderated mediation (IMM) was computed to assess the difference between the two conditional indirect effects. All regression coefficients reported in the results section are unstandardized (β) for sake of interpretability.

## 3. Results

### 3.1. Participants

A sample of 559 participants were recruited. The sample comprised 162 males (29%) and 397 females (71%), aged from 18 to 72 years (*mean* = 34.87, *SD* = 14.98) and a BMI ranging from 15.62 to 50.68 (*mean* = 23.11; *SD* = 4.15). Further details are reported in Table 1.

### 3.2. Preliminary Analysis

Before testing the main hypotheses, the prevalence of food addiction and bivariate associations among study variables was examined. In accordance with the YFAS 2.0 scoring procedure, out of the total sample, 69 (12.3%) participants had a diagnosis of FA, while 490 (87.7%) participants did not have a diagnosis of FA.

The correlation analyses showed that the psychological variables included in the moderated mediation model were positively associated, supporting hypothesis 1 (H1)—as shown in Table 2. Moreover, all correlation values remained below the critical threshold of |0.80| and permitting the continuation of the statistical analyses. As hypothesized, deficits in reflective functioning were positively associated with emotional eating (*r* = 0.278, *p* < 0.001), uncontrolled eating (*r* = 0.259, *p* < 0.001), and FA symptoms (*r* = 0.222, *p* < 0.001). Furthermore, consistent with the literature, variables related to dysfunctional eating behavior were positively associated with each other—with a moderate to strong effect size. Indeed, emotional eating was positively associated with uncontrolled eating (*r* = 0.721, *p* < 0.001), and FA symptoms (*r* = 0.485, *p* < 0.001). Lastly, uncontrolled eating was positively associated with FA symptoms (*r* = 0.505, *p* < 0.001). These preliminary findings confirmed the appropriateness of including all variables in the subsequent moderated mediation model.

**Table 1:** Samples descriptive statistics.

	Descriptives	
Age (M, *SD*)	34.87	14.98
BMI (M, *SD*)	23.11	4.15
Gender (*n*, *%*)		
Male	162	29%
Female	397	71%
Civil Status (*n*, *%*)		
Single	181	32.4%
In a relationship	210	37.6%
Married	136	24.3%
Separated/divorced	22	3.9%
Widowed	10	1.8%
Education (*n*, *%*)		
Middle school degree	37	6.6%
High school degree	220	39.4%
Bachelor degree	268	47.9%
Master/Ph.D.	34	6.1%
Work Status (*n*, *%*)		
Student	227	40.6%
Full-time worker	198	35.4%
Part-time worker	42	7.5%
Entrepreneurs	47	8.4%
Unemployed	11	2.0%
Retired	34	6.1%
BMI Class (*n*, *%*)		
Severely underweight (<16)	1	0.2%
Underweight (16–18.49)	37	6.6%
Normal weight (18.5–24.99)	387	69.2%
Overweight (25–29.99)	99	17.7%
Class I obesity (30–34.99)	29	5.2%
Class II obesity (35–39.99)	3	0.5%
Class III obesity (>40)	3	0.5%
ED Diagnosis (*n*, *%*)		
No ED	508	90.8%
Anorexia Nervosa	22	3.9%
Bulimia Nervosa	10	1.8%
Binge Eating Disorder	12	2.1%
ED No Otherwise Specified	7	1.3%

Note: M = mean; SD = standard deviation; *n* = number of individual in each category; % percentage.

**Table 2:** Descriptive statistics, correlations among variables.

		Descriptives				Correlations				
		M	SD	SK	K	1	2	3	4	5
1	(deficits in) Reflective functioning	2.63	3.28	1.66	2.82	-				
2	Emotional eating	6.30	2.59	0.39	−0.83	0.278	-			
3	Uncontrolled eating	17.50	5.74	0.64	−0.26	0.259	0.721	-		
4	YFAS2 Symptom Count	1.35	2.35	2.02	3.49	0.222	0.485	0.505	-	
5	Body Mass Index	23.11	4.15	1.77	6.11	0.062 §	0.273	0.204	0.289	-

Note: all correlations are statistically significant with *p* < 0.001, except for § *p* > 0.050 ns. M = mean, SD = standard deviation, SK = skewness, K = kurtosis.

### 3.3. MANOVA Results

**Figure 2:**
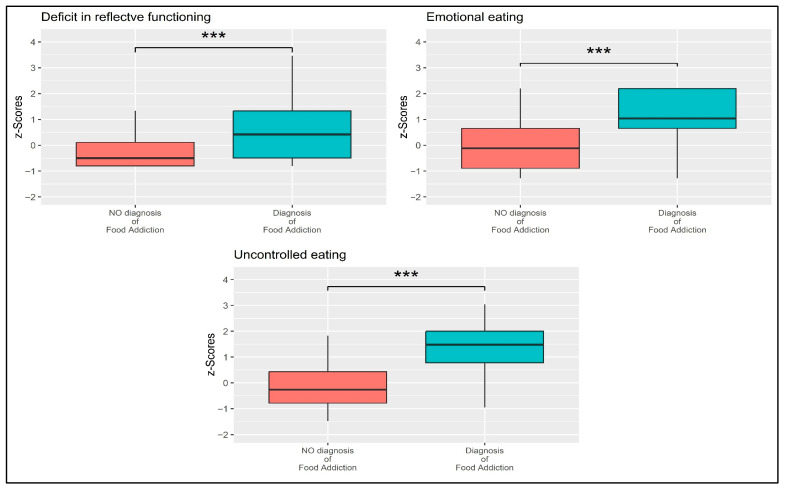
Boxplots showing the mean differences between participants without and with a diagnosis of FA. Note: *** = *p* < 0.001.

The raw score of each variable was almost normally distributed and their relationships were substantially linear. The Box’s *M* was statistically significant (*M* = 31.304, *F* = 5.132, *p* < 0.001)—however, it should be noted that MANOVA is robust to violations of assumptions [91,93]. Thus, considering these results, the MANOVA was performed. A statistically significant multivariate effect was found: Λ = 0.752, *F* = 60.998, *p* < 0.001; η^2^_p_ = 0.248 (large effect size). In line with Hypothesis 2 (H2), a statistically significant between groups difference for the RFQ8u scale [*t* = −5.775, *p* < 0.001; *g* = −0.742 (moderate effect size)], the emotional eating scale of the TEFRQ-18 [*t* = −10.786, *p* < 0.001; *g* = −1.385 (large effect size)], and the uncontrolled eating scale of the TEFRQ-18 [*t* = −12.742, *p* < 0.001; *g* = −1.636 (large effect size)]. Results are reported in Table 3 and Figure 2. Overall, these findings indicate that individuals with a diagnosis of FA exhibited significantly higher levels of reflective functioning deficits, emotional eating, and uncontrolled eating compared to those without FA diagnosis, with effect sizes ranging from moderate to large.

### 3.4. Moderated Mediation Analysis

**Figure 3:**
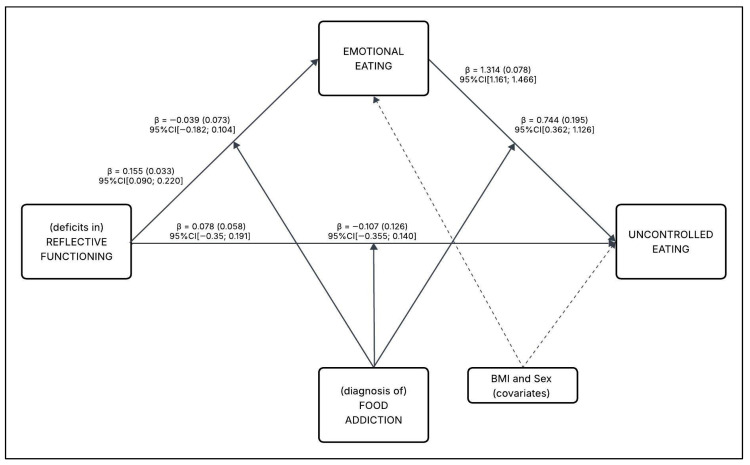
Path model with unstandardized regression coefficients, standard errors, and 95% confidence intervals.

To test the main hypothesis (H3), a conditional process analysis was conducted to examine whether the relationship between reflective functioning deficits and uncontrolled eating is mediated by emotional eating, and whether this mediation pathway is moderated by the presence of a FA diagnosis. The moderated mediation analysis (Figure 3) supported the hypothesized results, as detailed below and summarized in Table 4.

#### 3.4.1. Direct Effects and Main Associations

The direct paths in the model were first examined, controlling for covariates. As hypothesized, (deficits in) reflective functioning (X) were positively associated with emotional eating (M), *path a*: β = 0.155 (SE = 0.033), 95%CI [0.090; 0.220]. This indicates that greater deficits in reflective functioning were associated with higher levels of emotional eating. In addition, diagnosis of FA (W) was statistically associated (main effect) with emotional eating (M), *path v1*: β = 2.516 (SE = 0.431), 95%CI [1.669; 3.364]. This suggests that a FA diagnosis was associated with increased emotional eating, independent of reflective functioning.

Moreover, emotional eating (M) statistically positively predicted uncontrolled eating (Y), *path b*: β = 1.314 (SE = 0.078), 95%CI [1.161; 1.466]. This strong association confirms that higher emotional eating was linked to greater uncontrolled eating behaviors. However, diagnosis of FA (W) was not statistically associated (main effect) with uncontrolled eating (Y), *path v2*: β = −2.236 (SE = 1.753), 95%CI[−5.679; 1.207].

At the same time, controlling for emotional eating (M), (deficit in) reflective functioning (X) was no more statistically associated with uncontrolled eating (Y), *path c’*: β = 0.078 (SE = 0.058), 95%CI[−0.035; 0.191]—suggesting a complete mediation, wherein the effect of reflective functioning on uncontrolled eating was fully explained through emotional eating. The full model (including all direct effects, covariates, and interactions) explained 57.3% of the variance in uncontrolled eating (R^2^ = 0.573).

#### 3.4.2. Moderation Effects

Whether FA diagnosis moderated any of the pathways in the model was then tested. Out of the three interactions performed (*w*1, *w*2, *w*3), only the interaction between emotional eating (M) and diagnosis of FA (W) on uncontrolled eating (Y) was statistically significant (*path w*2: β = 0.744 (SE = 0.195), 95%CI [0.362; 1.126]). This indicates that the strength of the relationship between emotional eating and uncontrolled eating was dependent on whether a FA diagnosis was present. The non-significant interactions (w1 and w3) are detailed in Section 3.5.

#### 3.4.3. Conditional Indirect Effects

Finally, the indirect effects of reflective functioning on uncontrolled eating through emotional eating were examined separately for individuals with and without FA diagnosis. Considering the presence of a dichotomous moderator—namely, the diagnosis of FA (0 = absent vs. 1 = present)—the moderated mediation analysis yielded two distinct indirect effects [(deficit in) reflective functioning → emotional eating → uncontrolled eating], one for each level of the moderator.

Considering the overall model—thus, including also the non-statistically significant interactions (paths w1 and w3)—when the moderator was equal to 0 (=FA diagnosis: absent) the total indirect effect was: β = 0.204 (SE = 0.046), 95%CI [0.119; 0.300]. Conversely, when the moderator was equal to 1 (=FA diagnosis: endorsed) the total indirect effect was: β = 0.239 (SE = 0.125), 95%CI [0.006; 0.498]. The IMM test for the difference between both conditional indirect effects (including the three moderations—*w*1, *w*2, *w*3) of the overall model was equal to 0.035 (0.133), 95%CI[−0.214; 0.309]—meaning that no difference between the conditional indirect effects was observed because two out of three moderations were not statistically significant.

However, given that only one moderation was significant, the model was respecified to include only the statistically significant interaction (*path w2*). In this more parsimonious model, when the moderator was equal to 0 (=FA diagnosis: absent) the total indirect effect was: β = 0.262 (SE = 0.043), 95%CI [0.183; 0.353]. Conversely, when the moderator was equal to 1 (=FA diagnosis: endorsed) the total indirect effect was: β = 0.402 (SE = 0.069), 95%CI [0.276; 0.544]. The IMM of this respecified model was equal to 0.141 (0.043), 95%CI [0.060; 0.229], suggesting that the indirect effect was significantly stronger when the FA diagnosis was endorsed. In practical terms, the pathway from reflective functioning deficits to uncontrolled eating (via emotional eating) was amplified by approximately 53% in individuals with FA diagnosis compared to those without.

### 3.5. Probing Interaction

Considering the whole model, the simple slope analysis was used to deepen the moderation effect of the conditional process model presented in Figure 1 and Figure 3.

The moderation analyses for *w*1 and *w*3 examined the effect of FA diagnosis (W) as a moderator. Specifically, *w*1 tested the relationship from (deficits in) reflective functioning (X) to emotional eating (M), controlling for covariates, while *w*3 tested the relationship from X to uncontrolled eating (Y), controlling for M and covariates. Both interactions were not statistically significant (*w*1: β = −0.039, SE = 0.073, 95%CI [−0.182; 0.104]; *w*3: β = −0.107, SE = 0.126, 95%CI [−0.355; 0.140]) and were therefore not further examined. This indicates that the effects of (deficits in) reflective functioning on emotional eating and uncontrolled eating were not conditioned by FA diagnosis.

Conversely, the moderation analysis (*w*2) involved the moderating effect of the diagnosis of FA (W) on the relationship between emotional eating (M) and uncontrolled eating (Y), controlling for (deficit in) reflective functioning (X) and covariates: *β* = 0.744 (SE = 0.195) 95%CI [0.362; 1.126]. The change in explained variance (Δ*R*^2^) of the moderated mediation model due to this interaction was equal to 1.1% [ΔR^2^ = 0.011; *F* = 14.622, *p* < 0.001].

**Figure 4:**
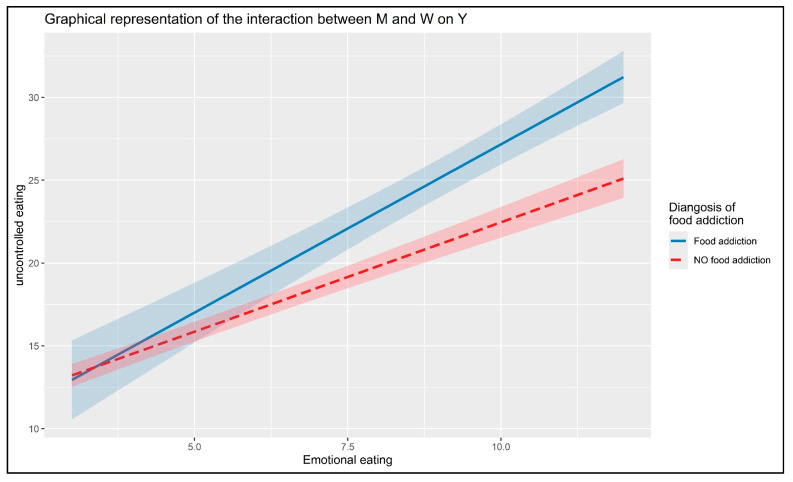
Interaction between emotional eating and diagnosis of food addiction of uncontrolled eating.

A deeper analysis was conducted on the two slopes. The simple slope analysis showed the effects at different levels of a binary moderator (0 = absent vs. 1 = endorsed)—Figure 4. On one hand, the simple slope analysis showed that the effect of emotional eating (M) on uncontrolled eating was minor when the diagnosis of FA was absent (moderator = 0): *β* = 1.314 (SE = 0.078), 95%CI [1.161; 1.466]. On the other hand, the effect was greater when the diagnosis of FA was present (moderator = 1): *β* = 2.057 (SE = 0.180), 95%CI [1.703; 2.412]). An additional statistical test was conducted to confirm the difference between the two slope coefficients. This direct comparison between the slopes indicated that the effect of emotional eating (M) on uncontrolled eating (Y) significantly differed as a function of FA diagnosis [*t* = 3.787, *p* < 0.001]—confirming once more a stronger association observed when the diagnosis of FA was present.

## 4. Discussion

In recent years, a growing number of studies have focused on the crucial role of psychological factors rooted in early life experiences—such as childhood maltreatment, attachment patterns, and deficits in reflective functioning—which may underlie the development of disordered eating behaviors [15,17]. At the same time, although still limited, a steadily increasing body of research has suggested that maladaptive eating behaviors may be intensified by the presence of a key factor like FA [5,49,103,104]. However, to date, no model has been tested that integrates all these variables while highlighting the strengthening role of FA.

The aim of the present study was therefore to address this gap in the literature by testing a moderated mediation model, in which deficits in reflective functioning were linked to uncontrolled eating behaviors through emotional eating—a dysfunctional internal regulation strategy. Furthermore, it was hypothesized that the presence of a FA diagnosis would moderate these relationships. Understanding these mechanisms can help to clarify the psychological processes underlying uncontrolled eating behaviors, which often stem from basic strategies for regulating internal states and may be exacerbated by conditions that are frequently overlooked or underestimated in clinical practice—such as a diagnosis of FA. Moreover, shedding light on the psychological dynamics associated with FA can contribute to the development of more effective interventions for individuals struggling with obesity and disordered eating behaviors related to overeating. In turn, this could inform the design of timely psychological treatments and encourage further exploration of their effects on other constructs within research settings.

### 4.1. Reflective Functioning and Emotional Eating

One of the main findings of this study was that deficits in reflective functioning were positively associated with emotional eating—although the strength of this association was modest. However, this result is fully consistent with the (still limited) existing literature. This finding replicates prior research [15] and supports the idea that individuals with deficits in mentalization tend to rely on external regulation strategies involving external objects—such as food.

Deficits in reflective functioning, often linked to traumatic childhood experiences, hinder the development of secure attachment and the ability to mentalize emotional states, particularly during stressful situations [15,105,106,107,108]. Under such conditions, individuals may shift from controlled mentalization to more automatic, limbic-driven responses [25,32,48,107,108], creating a need for external objects like food to manage distressing psychological states. Consequently, emotional eating becomes a compensatory response to this unmet need for internal self-regulation.

### 4.2. The Pathway from Emotional Eating to Uncontrolled Eating

Moreover, among the main findings of the study, the tested model confirmed the expected relationship between emotional eating and uncontrolled eating [5,8,55]. These results are in line with previous studies that have highlighted the role of emotional eating as a first strategy to externally regulate internal cognitive and emotional states [57,58,59,61] that could lead to more intense and dysregulated eating pattern [49,60].

It is important to note that this progression is particularly evident when considering the characteristics of the measurement tools used. The emotional eating scale of the TFEQ-18-R captures the tendency to turn to food in response to negative or uncontrollable emotions. In contrast, the uncontrolled eating scale measures the extent to which individuals engage in actual episodes of loss-of-control eating (not limited to binge eating but also including other eating behaviors such as grazing), which represent behaviors that are qualitatively and quantitatively more severe than emotional eating.

The present findings are particularly relevant when considering the specific characteristics of highly palatable and processed foods (HPFs)—foods high in sugar, fat, and salt—which are particularly implicated in FA symptoms as they may trigger addictive-like responses [69]. The synergistic effects of sugar and fat macronutrients activate reward pathways in ways that single macronutrients do not [109,110]. The hyperpalatable nature of these foods may be especially problematic for individuals with deficits in reflective functioning, as they may be less able to cognitively regulate their responses to food cues. Furthermore, the combination of high sugar and fat content can lead to rapid fluctuations in blood glucose and subsequent cravings, potentially exacerbating the cycle from emotional eating to uncontrolled eating behaviors [70]. This suggests that interventions targeting FA might benefit from addressing not only psychological mechanisms but also the specific nutritional composition of foods consumed.

### 4.3. The Amplifying Role of Food Addiction

Finally, the main finding of the study concerns the moderating role of FA diagnosis. The research partially confirmed the proposed hypothesis: FA diagnosis moderated the relationship between emotional eating and uncontrolled eating (starting from deficits in reflective functioning)—w2. Specifically, the presence of a FA diagnosis appeared to act as an amplifying factor in the enactment of maladaptive eating behaviors, originating from dysfunctional coping strategies (e.g., emotional eating) associated with the inability to self-regulate.

Findings suggest that individuals with a FA diagnosis were significantly more likely to engage in uncontrolled eating behaviors (e.g., grazing, binge eating). This was further supported by the slope analysis, which showed that participants with FA exhibited a stronger association—a steeper slope—between emotional eating and uncontrolled eating, indicating a higher intensity of behavioral dysregulation when this addictive component was present. Specifically, the interaction (w2) showed that, controlling for deficits in reflective functioning, emotional eating led to higher levels of uncontrolled eating; however, individuals who endorsed FA diagnosis exhibited uncontrolled eating scores that were 0.744 points higher compared to those who did not endorse this diagnosis.

### 4.4. Specificity of Food Addiction’s Moderating Role

Additionally, the results indicated that FA diagnosis did not significantly moderate either the relationship between deficits in reflective functioning and emotional eating (w1) or the relationship between deficits in reflective functioning and uncontrolled eating (w3). This lack of significance can be interpreted through several theoretical and methodological considerations that provide important insights into the specificity of FA’s moderating role within the proposed model.

From a theoretical perspective, deficits in reflective functioning likely represent a more fundamental and transdiagnostic vulnerability factor that operates independently of FA diagnosis. Being rooted in early caregiver relationships, reflective functioning influences eating behaviors through primitive and automatic mechanisms that do not appear to interact with specific food-related addictive characteristics. Impaired reflective functioning serves as a common prerequisite predisposing individuals to regulation strategies requiring external objects—both emotional eating and uncontrolled eating. This interpretation aligns with research suggesting that deficits in reflective functioning constitute a “fertile ground” for various forms of psychopathology [32], including eating disorders [15,51,53,111,112,113] and addictive behaviors [17,31,40,114,115].

From a methodological standpoint, the dichotomous nature of the moderator variable and relatively low FA prevalence (12.3%) may have limited statistical power for detecting subtle moderation effects. However, the significant w2 interaction demonstrated sufficient sensitivity to identify robust effects, supporting the premise that FA’s influence is most pronounced at specific transition points rather than uniformly across all pathways. These findings highlight FA as a specific amplifying factor rather than a general moderator, emphasizing the temporal and mechanistic specificity of addictive processes in eating behavior research.

In summary, FA appeared to act as a moderator only at specific stages of the dysfunctional eating continuum—specifically during the transition from emotional eating to uncontrolled eating (w2), rather than in initial stages originating from reflective functioning deficits (w1 and w3). This pattern aligns with theoretical models suggesting that addictive characteristics emerge when compensatory behaviors fail to provide adequate emotional regulation, requiring more intense strategies [49,60]. While FA captures core addiction-related aspects [80,89], these elements may not directly condition fundamental mechanisms translating reflective functioning deficits into stronger use of external regulation strategies (emotional eating), but rather amplify their intensity and consequent behaviors. Therefore, FA could play a specific key role as a promoting factor and/or reinforcement between the use of external regulators in response to negative emotions and the engagement in dysfunctional and uncontrolled eating behavior [17].

This finding aligns with the theoretical premise of the study, which posits that deficits in reflective functioning may serve as a predisposing risk factor for the development of dysfunctional facets related to eating behavior (such as emotional eating). When an individual lacks the ability to regulate internal emotional states, they may seek gratification through external objects—such as HPFs that are strongly associated with food addiction [116,117,118,119,120]. When emotional food intake fails to achieve the desired internal regulation, the behavior may escalate, overwhelming the individual and culminating in uncontrolled eating—an outcome that is particularly intensified in the presence of a FA diagnosis.

### 4.5. Neurobiological Considerations and Weight Implications

The potential role of neurobiological factors should also be considered Deficits in reflective functioning have been associated with altered prefrontal cortex activity, particularly in regions implicated in executive control and emotion regulation [108].

When these top-down regulatory mechanisms are compromised, individuals may become more reliant on limbic-driven responses to emotional distress, including maladaptive behaviors such as food-seeking [72]. The presence of a FA diagnosis may further exacerbate this dysregulation by sensitizing reward pathways and diminishing the capacity to resist food-related cues [116], thereby fostering self-reinforcing cycles of problematic eating.

This mechanism may have important implications for weight management. The pathway from deficits in reflective functioning to uncontrolled eating—mediated by emotional eating and amplified by FA—represents a psychological risk profile that could contribute to weight gain over time. Prospective studies have demonstrated that emotional eating consistently predicts weight gain in adults, with individuals showing higher emotional eating scores experiencing greater increases in BMI and waist circumference over time [121,122]. Notably, the stronger effect observed among individuals with FA diagnosis (considering only the respecified model, β = 0.262 vs. β = 0.402) suggests that this subgroup may be particularly vulnerable to adverse weight-related outcomes [122], as FA symptoms have been linked to impaired control over eating behavior and greater psychological distress [123]. However, given the cross-sectional nature of this study, causal inferences about weight trajectories cannot be drawn. Future longitudinal research is therefore warranted to clarify how these psychological pathways may contribute to weight changes across time.

Lastly, the controversy surrounding FA and its role in obesity development is particularly relevant to these findings. HPFs [68,124] are particularly activating for brain circuits, as evidenced by research showing their role in FA symptoms [65,66,67]. In pediatric populations, the concern is particularly acute as children’s developing brains may be more susceptible to the rewarding properties of these foods [125]. Children with early trauma or attachment difficulties—conditions associated with impaired reflective functioning [15,17,25]—may be especially vulnerable to developing problematic relationships with food [126]. However, the complexity of neurobiological and environmental mechanisms warrants caution in interpretation and highlights the need for further investigation. Future studies should continue to explore the interplay between HPFs consumption, psychological vulnerabilities, and long-term health outcomes across diverse populations.

### 4.6. Strength, Limitations, and Future Research

This study is not without limitations. First, due to the cross-sectional design, causal inferences cannot be drawn. However, the research was grounded in a well-established theoretical framework, which informed both the formulation of hypotheses and the structure of the analytical model. It is also worth noting that some variables examined—particularly those linked to developmental history and early-life experiences—are inherently stable over time and, within the constraints of statistical modeling, should be considered as predictors [77,127].

Second, the study relied exclusively on self-report questionnaires, without the inclusion of clinical interviews or behavioral observations, which would offer a more nuanced assessment of reflective functioning deficits and a more accurate diagnosis of FA. The reliance on self-report measures may introduce common method variance and recall bias, particularly for retrospective assessments. Additionally, the snowball sampling approach and online administration may have introduced selection and response biases compared to randomized sampling and clinical assessments. Nonetheless, all questionnaires employed—such as the YFAS 2.0—are psychometrically sound and widely recognized for their validity and reliability in capturing the constructs under investigation.

Third, the relatively narrow scope of variables considered represents a limitation. Constructs such as impulsivity, difficulties in emotional regulation, and attachment insecurity—known to be associated with disordered eating and addictive behaviors—were not included but warrant attention in subsequent studies. Future studies may also consider relational and social factors (e.g., social support) contributing to mental health outcomes [128,129]. Future research should also incorporate physiological measures such as cortisol levels, neuroimaging data examining prefrontal-limbic connectivity, and metabolic markers to examine biological mechanisms underlying these psychological pathways. Additionally, genetic factors related to dopamine functioning and food reward sensitivity warrant investigation.

Fourth, the sample showed a slight gender imbalance, with female participants being overrepresented. This highlights the importance of considering gender and ethnicity-related factors in future research. Although the sample size was adequate and diverse, the findings can only be generalized to the Italian population and may be subject to sampling biases. Future studies should aim to recruit more representative samples or replicate the findings in clinical populations, such as individuals with diagnosed eating disorders or obesity.

Fifth, as noted in previous studies, the use of two scales targeting food-related constructs (YFAS 2.0 and the UE scale from the TFEQ-18-R) introduces the possibility of semantic overlap, which could potentially inflate correlations. However, this risk appears minimal in the present study, as observed correlations remained below critical thresholds [91,130,131], and the instruments measure conceptually distinct dimensions of eating behavior. Specifically, the YFAS 2.0 assesses hallmark symptoms of FA—including tolerance, withdrawal, and cravings—whereas the uncontrolled eating scale captures patterns of dysregulated eating, such as excessive food intake and impaired control over eating [73,132]. Furthermore, FA is not limited to overt episodes of binge eating or overeating; it can also manifest in more subtle and persistent patterns, such as grazing [6,7,133].

Finally, although the percentage of participants with FA diagnosis is consistent with available data for the general population, it remains low (12.3%; 69 participants). While sample size was calculated a priori using Monte Carlo simulation, this limited number may have influenced results. Future studies could consider increasing sample size to achieve greater power for participants with FA diagnosis. Despite these limitations, the theoretical grounding, validated instruments, and robust statistical approach support the reliability of conclusions within these methodological constraints.

However, the study presents several noteworthy strengths. Most importantly, it is anchored in a coherent and well-articulated theoretical model, which informed both the design and the statistical approach. The use of validated instruments and a relatively large, heterogeneous sample adds further robustness to the findings. To the best of our knowledge, this is the first study to examine the interplay between reflective functioning and disordered eating behaviors while specifically highlighting the moderating role of FA. Finally, by emphasizing the contribution of psychological processes to patterns of overeating and reduced dietary control, the findings resonate with foundational principles of contemporary therapeutic approaches—such as mentalization-based treatment (MBT) [44,134,135].

### 4.7. Clinical Implications

This study offers important contributions for both scientific research and clinical application. On the clinical front, the findings support the integration of therapeutic strategies grounded in contemporary, evidence-based psychotherapies—such as MBT [136,137,138]. Specifically, the results indicate that clinical interventions might benefit from focusing on strengthening individuals’ capacity to manage the emotional and behavioral outcomes associated with deficits in reflective functioning. Addressing maladaptive cognitive patterns, difficulties in emotion regulation, and dysfunctional behaviors may foster greater psychological well-being and reduce the incidence of disordered eating behaviors linked to FA. These interventions could be particularly effective when integrated with structured treatment protocols designed for such conditions [44,134,135,139,140,141,142,143,144].

Building on these therapeutic approaches, the findings suggest several specific intervention targets. The moderating role of FA diagnosis indicates that treatments should be stratified based on individual risk profiles, with individuals showing FA symptoms requiring interventions that incorporate craving management and trigger identification strategies. The stronger association between emotional eating and uncontrolled eating observed in individuals with FA diagnosis (β = 2.057 vs. β = 1.314) highlights the importance of early intervention targeting emotional eating patterns to prevent progression to more severe dysregulated behaviors. Furthermore, the results support multi-level interventions addressing underlying psychological processes, behavioral patterns, and environmental factors, with prevention programs focusing on developing emotional regulation skills before problematic patterns become entrenched.

From a research standpoint, the study deepens current knowledge by empirically supporting the association between reflective functioning and FA [17]. This contributes to a growing body of literature and lays the groundwork for future investigations exploring these psychological mechanisms. Moreover, the findings highlight the critical role played by deficits in reflective functioning, emotional eating, and the presence of FA diagnosis in mediating and moderating the path to uncontrolled eating behaviors. By clarifying these associations, the study provides clinicians with a conceptual basis for developing focused interventions aimed at individuals displaying dysregulated eating behaviors.

## 5. Conclusions

This research advances the understanding of maladaptive eating patterns—specifically emotional eating, uncontrolled eating, and FA—in relation to impairments in reflective functioning. As far as we are aware, it represents the first attempt to explore this specific pathway, particularly highlighting the moderating influence of FA. These insights may open new directions for the development of targeted interventions addressing disordered eating behaviors connected to FA symptoms and diagnosis. The results suggest that deficits in reflective functioning are directly linked to problematic eating behaviors, and that this relationship is further moderated by the presence of FA diagnosis.

## Figures and Tables

**Table 3:** MANOVA results.

	No Diagnosis of FA (*n* = 490)		Diagnosis of FA (*n* = 69)				
	M	SD	M	SD	*t*	*p*-Value	*g*
(deficits in) Reflective functioning	2.34	3.03	4.71	4.18	−5.775	<0.001	−0.742
Emotional eating	5.90	2.32	9.18	2.61	−10.786	<0.001	−1.385
Uncontrolled eating	16.48	4.81	24.76	6.56	−12.742	<0.001	−1.636

Note: M = mean; *SD* = Standard deviation; *t* = *t*-test; *g* = Hedges, g.

**Table 4:** Summary of standardized (β*) and unstandardized (β) parameter estimates with 95% Confidence Intervals of the tested model (Figure 3).

		β*	β (SE)	95%CI [L-U]	z-Value	*p*-Value	*R* ^2^
(deficits in) reflective functioning (X) → Emotional eating (M)	a	0.197	0.155 (0.033)	[0.090; 0.220]	4.675	<0.001	0.271
Diagnosis of FA (W) → Emotional eating (M)	v1	0.319	2.516 (0.431)	[1.669; 3.364]	5.332	<0.001	
(deficits in) reflective functioning (X) * Diagnosis of FA (W) → Emotional eating (M)	w1 (X*W → M)	−0.032	−0.039 (0.073)	[−0.182; 0.104]	−0.612	0.541	
BMI → Emotional eating (M)		0.228	0.142 (0.024)	[0.096; 0.189]	5.583	<0.001	
Sex → Emotional eating (M)		0.177	1.013 (0.214)	[0.593; 1.433]	4.945	<0.001	
							
Emotional eating (M) → Uncontrolled eating (Y)	b	0.606	1.314 (0.078)	[1.161; 1.466]	17.548	<0.001	0.574
Diagnosis of FA (W) → Uncontrolled eating (Y)	v2	−0.131	−2.236 (1.753)	[−5.679; 1.207]	−1.262	0.207	
							
Emotional eating (M) * Diagnosis of FA (W) → Uncontrolled eating (Y)	w2 (M*W → Y)	0.418	0.744 (0.195)	[0.362; 1.126]	4.132	<0.001	
							
(deficits in) reflective functioning (X) * Diagnosis of FA (W) → Uncontrolled eating (Y)	w3 (X*W → Y)	−0.041	−0.107 (0.126)	[−0.355; 0.140]	−0.744	0.457	
							
(deficits in) reflective functioning (X) → Uncontrolled eating (Y)	c’	0.045	0.078 (0.058)	[−0.035; 0.191]	1.135	0.256	
BMI → Uncontrolled eating (Y)		−0.034	−0.046 (0.042)	[−0.128; 0.035]	−1.098	0.272	
Sex → Uncontrolled eating (Y)		−0.067	−0.831 (0.370)	[−1.558; −0.103]	−2.213	0.027	
							
Indirect effect of X on Y via M (FA diagnosis = absent)	a*b; FA = 0	0.119	0.204 (0.046)	[0.117; 0.299]	4.489	<0.001	
Indirect effect of X on Y via M (FA diagnosis = endorsed)	a*b; FA = 1	0.169	0.239 (0.118)	[0.013; 0.474]	2.069	0.044	
Total effect of X on Y (FA diagnosis = absent)		0.165	0.281 (0.072)	[0.143; 0.426]	3.918	<0.001	
Total effect of X on Y (FA diagnosis = endorsed)		0.173	0.209 (0.178)	[−0.131; 0.570]	1.177	0.239	

Note: β* = standardized beta; β = unstandardized beta; SE = standard error; 95%CI = 95% confidence intervals (lower/upper) for the unstandardized beta; *R*^2^ = explained variance; Emotional eating = TFEQ-18-R emotional eating scale; Uncontrolled eating = TFEQ-18-R uncontrolled eating scale; (deficits in) reflective functioning = RFQ-8 Uncertainty scale; Diagnosis of FA = endorsement of a FA diagnosis using YFAS2.0 (coded: 0 = absence vs. 1 = endorsed).

## Data Availability

Data are available on a reasonable request due to privacy and ethical restrictions.
